# A functional SNP rs1892901 in *FOSL1* is associated with gastric cancer in Chinese population

**DOI:** 10.1038/srep41737

**Published:** 2017-02-07

**Authors:** Wenjie Liu, Tian Tian, Li Liu, Jiangbo Du, Yayun Gu, Na Qin, Caiwang Yan, Zhaoming Wang, Juncheng Dai, Zhining Fan

**Affiliations:** 1 Digestive Endoscopy Center, The First Affiliated Hospital of Nanjing Medical University and Jiangsu Province Hospital, Nanjing 210029, China; 2 Department of Epidemiology and Biostatistics, Collaborative Innovation Center For Cancer Personalized Medicine, School of Public Health, Nanjing Medical University, Nanjing 211166, China; 3 Department of Epidemiology and Biostatistics, School of Public Health, Nantong University, Nantong 226019, China; 4 Jiangsu Key Lab of Cancer Biomarkers, Prevention and Treatment, Collaborative Innovation Center of Cancer Medicine, Nanjing Medical University, Nanjing 211166, China

## Abstract

*FOSL1* (FOS like antigen 1) is one kind of proto-oncogene, and may play a vital role in carcinogenesis of multiple cancers. However, studies about the relationship between SNPs in *FOSL1* and gastric cancer are still lacking. Thus, we investigated the association of seven SNPs in *FOSL1* with gastric cancer using case-control design in a two-stage strategy (Screening stage: 1,140 gastric cancer cases and 1,547 controls; Replication stage: 1,006 cases and 2,273 controls). We found that rs1892901 was significantly associated with increased risk of gastric cancer in additive model (adjusted OR = 1.25, 95%CI: 1.06–1.47, *P* = 0.008) in first stage. Following replication results revealed that the relationship between rs1892901 and gastric cancer risk was consistent with our primary results. *In silico* analysis showed that rs1892901 might alter multiple regulatory motifs, disturb protein binding, and affect the expression of *FOSL1* and other important gastric cancer-related genes such as *EGR1, CHD, EP300, FOS, JUN* and *FOSL2.* Our findings indicated that functional SNP rs1892901 in *FOSL1* might affect the expression of *FOSL1*, and ultimately increase the risk of gastric cancer. Further functional studies and large-scale population studies are warranted to confirm our findings.

The incidence and mortality of gastric cancer have been steadily decreasing in the past decades throughout the world, especially in the developed countries^1^,^2^. However, in China, gastric cancer is still the second most common cancer with an estimated 679,100 new cases and the second leading cause of cancer deaths with an estimate of 498,000 deaths in 2015^3^. Gastric cancer is the result of complex interactions of environmental exposures (including *Helicobacter pylori* infection, cigarette smoking, unhealthy diet, obesity, etc) and genetic factors^4^. A growing number of genotyping methods and strategies have been applied to explore the genetic risk of gastric cancer. Although association studies based on high-throughput technology (e.g., genome-wide association study) could identify numerous gastric cancer susceptibility SNPs (single nucleotide polymorphisms), the findings could explain only a small fraction of gastric cancer heritability. Therefore, conventional candidate-gene based strategy is still a useful tool to detect crucial variants of gastric cancer.

FOS like antigen 1 (*FOSL1*) is a member of the Fos gene family that plays important roles in cell proliferation, differentiation, transformation and metastasis^5^,^6^. Proteins of the JUN family and FOSL1 can form activator protein-1 complexes (transcription factor AP-1), which are responsible for misregulation in carcinogenesis. AP-1 is a major mediator of transformation by Ras, and FOSL1 is the predominant protein which contributes to the activity of AP-1^7^,^8^. It has been reported that FOSL1 was associated with a more malignant phenotype and might play a pivotal role in cancer progression^9^,^10^. FOSL1 can be regulated transcriptionally and post-translationally; besides, it can positively regulate transcription as well^11^. It has been confirmed that, *FOSL*1 is a kind of proto-oncogene, and elevated expression of FOSL1 is detected in multiple human carcinomas, including colon adenocarcinoma, ovarian cancer, breast cancer, head and neck, lung and esophageal squamous cell carcinoma, etc^9^,^12^. In 2015, He *et al*. suggested that FOSL1 was upregulated in gastric cancer tissues and might affect the PI3K/Akt and p53 signaling pathway in gastric cancer^6^. Thus, FOSL1 may influence the occurrence and development of multiple types of cancers including gastric cancer. Nevertheless, so far there are limited studies focusing on the relationship of genetic variants in *FOSL1* with gastric cancer.

In this study, we hypothesized that the SNPs in *FOSL1* gene might play an important role in the carcinogenesis and progression of gastric cancer. Thus, we investigated the association of seven SNPs in *FOSL1* gene with gastric cancer in a two-stage case-control study in Chinese (Screening stage: 1,140 gastric cancer cases and 1,547 controls; Replication stage: 1,006 cases and 2,273 controls).

## Results

As shown in Supplementary Table 1, the distributions of age, gender, drinking and smoking status between cases and controls for the Screening stage were comparable (*P* > 0.05). The detailed distributions of the characteristics mentioned above between cases and controls for the Replication stage were reported previously^13^. Genotyping rates for all of the seven SNPs in *FOSL1* were more than 99% in the Screening stage. The observed genotype frequency for each SNP was in agreement with Hardy-Weinberg equilibrium in controls (*P* > 0.05). Among the seven SNPs, rs1892901 was significantly related to the gastric cancer risk in additive model (adjusted OR = 1.25, 95%CI: 1.06–1.47, *P* = 0.008, *P* for *FDR* = 0.056). However, no significant associations were observed for the remaining six SNPs (rs637571, rs10791830, rs653914, rs614520, rs10896065, rs7940700) in additive model (Table 1).

The genotype distributions of rs1892901 between cases and controls in two stages were shown in Table 2. In the Screening stage, after adjustment for age, gender, drinking status and smoking status, presence of AA genotype was significantly associated with the increased risk of gastric cancer (adjusted OR (odds ratio) = 2.13, 95% CI (confidence interval): 1.11–4.06, *P* = 0.022) in comparison with presence of GG genotype of rs1892901. However, presence of GA genotype were not related to gastric cancer risk (adjusted OR = 1.19, 95%CI: 0.99–1.43, *P* = 0.067). In the following replication stage (1,006 cases and 2,273 controls), the relationship between rs1892901 and gastric cancer risk was consistent with our primary results (adjusted OR = 1.22, 95%CI: 1.04–1.44, *P* = 0.016, Table 2). The combined results of the two stages further showed rs1892901 was related to gastric cancer risk in additive model (adjusted OR = 1.24, 95%CI: 1.10–1.39, *P* < 0.001, Table 2). Compared with presence of GG of rs1892901, presence of GA and AA were both significantly associated with the increased risk of gastric cancer (Table 2, adjusted OR = 1.18, 95%CI: 1.04–1.35, *P* = 0.013 for GA; adjusted OR = 2.04, 95%CI: 1.30–3.20, *P* = 0.002 for AA). The risks of gastric cancer associated with rs1892901 were similar for both stages (*P*_heterogeneity_ > 0.05).

In order to further explore the associations of rs1892901 with gastric cancer risk, we conducted the stratified analyses within subgroups according to age, gender, smoking and drinking status (Supplementary Table 2). No significant heterogeneity was identified between different subgroups for SNP rs1892901 (*P*_*heterogeneity*_ > 0.05). Rs637571 (A allele) was found to be a protective factor for gastric cancer development, and the association with decreased gastric cancer risk achieved a boundary significance (*P* = 0.094). Thus, we investigated the interaction of rs637571 with individuals’ demographics on gastric cancer risk. However, no significant multiplicative interaction was present (Supplementary Table 3).

**Figure 1:**
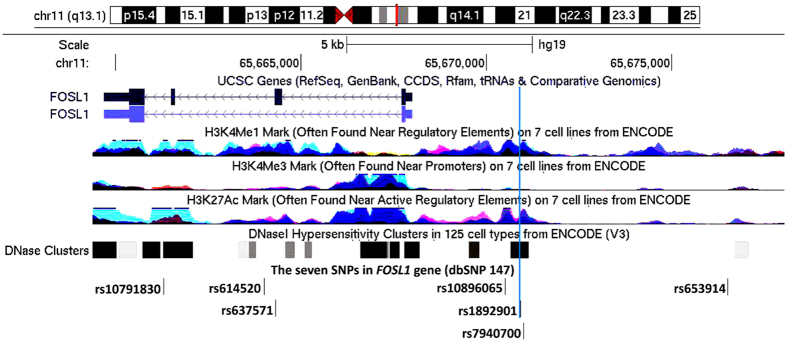
Functional annotation in proximity to SNP rs1892901 in FOSL1 location. CHIP-seq tracks for promoter histone marks (H3K4Me3) and enhancer histone marks (H3K4Me1, H3K27Ac) in seven cell lines (GM12878, H1-hESC, HSMM, HUVEC, K562, NHEK, and NHLF cells) are present along with DNase hypersensitivity tracks from ENCODE data through UCSC website. The light blue, long vertical line indicates the position of SNP rs1892901.

Based on the UCSC Genome Bioinformatics website (http://genome.ucsc.edu/), we annotated rs1892901 in regulatory elements which catalogued in Encyclopedia of DNA Elements (ENCODE) project. As shown in Fig. 1, rs1892901 is situated near the enhancer elements (H3K4Me1 mark) and also near the active promoter elements (H3K27Ac mark) on seven cell lines from ENCODE, which indicated that rs1892901 was probably involved in the regulation of gene expression. Besides, rs1892901 fell into DNase I Hypersensitivity peaks in 125 cell types from ENCODE, which suggested a possible mechanism for the effect on gastric cancer risk.

## Discussion

In the present study, we systematically evaluated the relationships of tagging SNPs in *FOSL1* gene with gastric cancer risk in a two-stage case-control study in Chinese (Screening stage and Replication stage). The SNP rs1892901 was identified to be significantly associated with the increased risk of gastric cancer in both stages. Additionally, *in silico* analysis indicated that rs1892901 might affect transcriptional regulation and expression of certain important gastric cancer-related genes.

FOSL1 (also known as FRA-1) is a member of the Fos transcription factor family (including c-Fos, FosB, Fra-1 and Fra-2) that is highly expressed in multiple tumors^14^. FOSL1 and the JUN family (c-Jun, JunB, and JunD) can form activator protein-1 (AP-1) complexes that have been confirmed to mediate various biological processes including cell proliferation, cell differentiation, tumorigenesis, and neoplastic transformation^15^,^16^,^17^. It has been reported that both Fos family and JUN family could function as oncogenic transcription factors^18^, while the increased expression of FOSL1 might be more predictive of oncogenic activity^19^. Dysregulation of FOSL1 has been discovered in multiple cancers and tumor cell lines including colorectal cancer, head and neck squamous cell carcinoma, breast cancer, etc. Besides, frequent over-expression of FOSL1 is identified in squamous cell carcinomas of the stomach and esophagus^19^,^20^. It has been reported that FOSL1 could regulate several cancer-related genes and microRNAs, including *MMP-1, MMP-9, CD44*, E2H2, microRNA-195 and microRNA-34a^11^,^21^,^22^,^23^. In addition, He *et al*. found that the expression of FOSL1 was higher in the gastric cancer samples in comparison with the adjacent non-cancerous tissues, and over-expression of FOSL1 could influence the expression of vital cancer-related genes including *PI3K, Akt, MDM2*, and *p53*^6^. Thus, FOSL1 was up-regulated in gastric cancer and might affect the expression of certain important gastric cancer-related genes, and further affect the development of gastric cancer.

So far no association study has been conducted to explore the relationship of polymorphisms in *FOSL1* with gastric cancer risk. Our study revealed that SNP rs1892901 in *FOSL1* was associated with the increased risk of gastric cancer. *In silico* analysis showed that SNP rs1892901 falls into DNase I Hypersensitivity peaks in 125 cell types from ENCODE, which suggested that there was a potential mechanism for the effect on gastric cancer risk. Besides, rs1892901 is situated near the enhancer elements (H3K4Me1 mark) and the active promoter elements (H3K27Ac mark) in 7 cell lines from ENCODE, which might suggest that this SNP is probably involved in the regulation of gene expression. In addition, according to SNPinfo (http://snpinfo.niehs.nih.gov/)^24^, an online tool, rs1892901 was confirmed to be a potential transcription factor binding site. Moreover, based on another web-based analysis tool, RegulomeDB (http://www.regulomedb.org/), the Regulome DB score of rs1892901 was 2b, which means this SNP is likely to alter multiple regulatory motifs and disturb protein binding activities. Rs1892901 might affect the binding of multiple important transcription-related proteins such as EGR1, CHD, EP300, FOS, JUN, FOSL1, and FOSL2. It has been reported that there are some relations between the proteins mentioned above and gastric cancer^6^,^25^,^26^,^27^,^28^. Among these proteins, the genes encoding FOS, FOSL1, and FOSL2 are members of *Fos* gene family, and the gene encoding JUN belongs to the JUN family. As mentioned above, the leucine zipper proteins encoded by *Fos* genes and proteins of genes of *JUN* family members could form the transcription factor complex AP-1. In 2011, Luo *et al*. revealed that negatively regulated AP-1 activity could lead to the decreasing mRNA level of cyclin D1 in gastric cancer cells^27^, which indicated that AP-1 might be involved in proliferation inhibition in gastric cancer. Besides, in 2015, Xia *et al*. proposed that tumor promoter-induced matrix metalloproteinase-9 (MMP-9) could be inhibited through suppression of AP-1 activity in gastric cancer cells^29^. Totally, SNP rs1892901 may influence the expression of FOSL1 and other gastric cancer-related genes on the basis of some potential mechanisms (including modifying DNA methylation and transcription factor response elements), and ultimately affect the occurrence of gastric cancer. However, the functional evidence for rs1892901 in *FOSL1* was based on *in silico* analysis. Further well-designed functional studies are warranted to confirm our findings.

In summary, our study revealed that functional SNP rs1892901 in *FOSL1* might affect the expression of *FOSL1* and other gastric cancer-related genes, and ultimately modify the gastric cancer risk in Chinese. Our results could be a clue for further functional studies to reveal the underlying biological mechanisms in gastric carcinogenesis.

## Materials and Methods

### Ethics Statement

All aspects of our study were approved by the Institutional Review Board of Nanjing Medical University (FWA00001501), and the study was carried out according to the principles of the Declaration of Helsinki. The design and the procedure of current study involving human individuals were described in a research protocol. Written informed consent was obtained from each participant before the start of the study.

### Study subjects

The recruitment of subjects for screening stage in this study was described previously^30^. Briefly, gastric cancer cases were consecutively recruited from Jiangsu Province, Eastern China. All of the gastric cancer cases were histopathologically confirmed, and those with any other type of cancer or having received chemotherapy or radiotherapy were eliminated. Totally, 1,140 gastric cancer cases were included in this study. And a total of 1,547 cancer-free controls were randomly selected from the individuals in a screening program for non-infectious diseases in Jiangsu province. The controls were frequency-matched to gastric cancer cases for sex, age, and regions (city of residence). Each participant was asked to answer the questions from a standard questionnaire, and donate about 5 ml venous blood sample. Individuals who smoked at least once per day for more than one year were considered to be smokers. And individuals who drank equal to or more than twice a week for at least one year were defined as drinkers. For Replication stage, we analyzed the relationship of positive SNPs in *FOSL1* (identified according to the Screening stage) and gastric cancer risk based on a published Chinese gastric cancer GWAS (including 1,006 gastric cancer cases and 2,273 controls)^8^, which was conducted using Affymetrix Genome-Wide Human SNP Array 6.0 chips.

### Selection and genotyping assays of polymorphisms

Common SNPs (minor allele frequency, MAF ≥ 0.05) were screened in *FOSL1* gene regions (including 10-kb up-stream region of this gene) in Chinese Han population (CHB) on the basis of the HapMap SNP database (phase II + III Feb 09, on NCBI B36 assembly, dbSNP b126). According to the above mentioned criteria, a total of 10 SNPs were identified. Based on an analysis website for SNP function prediction, SNPinfo Web Server (http://snpinfo.niehs.nih.gov/), four potentially functional SNPs (rs10896065, rs1892901, and rs637571, and rs7940700) were indicated. Linkage disequilibrium (LD) analysis with an r^2^ threshold of 0.80 was further applied to filter the tagging SNPs in 10 SNPs mentioned above (the potentially functional SNPs were prioritized in the final selection). As a result, seven SNPs were selected in the current study, including rs1892901 (G > A), rs637571 (G > A), rs10791830 (G > A), rs653914 (A > G), rs614520 (A > G), rs10896065 (G > A), rs7940700 (G > A).

Genotyping for the discovery stage was carried out using Illumina Infinium^®^ BeadChip (Illumina inc.), GenTrain version 1.0 clustering algorithm implemented in GenomeStudio V2011.1 (Illumina inc.) was used to call the clusters. Technicians were blinded to the case or control status of participants.

### Statistical analyses

The chi-square (*χ*^*2*^) test was applied to analyze distribution differences of each variable between cases and controls, including demographic characteristics, selected variables and genotypes. The goodness-of-fit *χ*^*2*^ was performed to test the deviation of genotype distribution for each SNP from the Hardy-Weinberg equilibrium. Logistic regression analyses were used to compute ORs and their 95% CIs for the relationships of the genotypes to risk of gastric cancer. Covariates for adjustment in this study included age, sex, smoking and drinking. BH-FDR procedure (Benjamini-Hochberg false discovery rate) was used as multiple testing to control type-I error rate^31^. The *χ*^*2*^- based Q-test was applied to test the heterogeneity between corresponding subgroups. All tests were two-sided and the significance level was set at *P* ≤ 0.05. All analyses were performed using R software (version 3.1.1, The R Foundation for Statistical Computing, http://www.cran.r-project.org/).

### *In silico* analysis

We annotated the promising SNPs in regulatory elements cataloged in Encyclopedia of DNA Elements (ENCODE) project according to UCSC Genome Bioinformatics website (http://genome.ucsc.edu/). We analyzed the data of H3K4Me3, H3K27Ac, and H3K4Me1 Mark on seven cell lines including H1-hESC, GM12878, K562, HUVEC, HSMM, NHLF, and NHEK cells, and examined DNaseI Hypersensitivity (DNaseI HS) data in 125 cell types.

## Additional Information

**How to cite this article:** Liu, W. *et al*. A functional SNP rs1892901 in *FOSL1* is associated with gastric cancer in Chinese population. *Sci. Rep.* **7**, 41737; doi: 10.1038/srep41737 (2017).

**Publisher's note:** Springer Nature remains neutral with regard to jurisdictional claims in published maps and institutional affiliations.

## Supplementary Material

Supplementary Tables

## Figures and Tables

**Table 1:** Primary information of 7 SNPs in *FOSL1* gene.

SNP	Location	Alleles^a^	Cases^b^ (N = 1,140)	Controls^b^ (N = 1,547)	MAF^c^	HWE^d^	Genotyping rate (%)	Additive model^e^	
								OR (95%CI)	*P*
rs1892901	5′ near gene	G/A	842/275/23	1199/330/16	0.141/0.117	0.221	99.9	**1.25 (1.06–1.47)**	**0.008**
rs637571	Intron	G/A	712/378/47	918/547/76	0.208/0.227	0.664	99.7	0.89 (0.78–1.02)	0.094
rs10791830	Intron	G/A	378/565/194	549/738/255	0.419/0.405	0.792	99.7	1.06 (0.95–1.19)	0.272
rs653914	5′ near gene	A/G	307/561/270	388/781/376	0.484/0.496	0.684	99.9	0.95 (0.85–1.06)	0.349
rs614520	Intron	A/G	717/369/54	969/517/60	0.209/0.206	0.437	100.0	1.02 (0.89–1.16)	0.812
rs10896065	5′ near gene	G/A	819/289/31	1105/409/30	0.154/0.152	0.323	99.9	1.02 (0.87–1.18)	0.831
rs7940700	5′ near gene	G/A	820/288/31	1105/408/30	0.154/0.152	0.322	99.8	1.02 (0.87–1.18)	0.848

^a^Major/minor allele;

^b^Major homozygote/heterozygote/Rare homozygote between cases and controls;

^c^Minor allele frequency between cases/controls;

^d^Hardy-Weinberg equilibrium test among controls;

^e^Logistic regression with adjustment for age, sex, smoking and drinking status.

**Table 2:** Associations between SNP rs1892901 in *FOSL1* gene and gastric cancer risk.

Genotype or genetic model a	Screening stage				Replication stage		Combined^b^	
	Cases/Controls	Adjusted OR (95% CI)^c^	*P*^c^	Cases/Controls	Adjusted OR (95% CI)^c^	*P*^c^	Adjusted OR (95% CI)^c^	*P*^c^
GG	842/1199	1.00		759/1786	1.00		1.00	
GA	275/330	1.19 (0.99–1.43)	0.067	227/463	1.17 (0.97–1.41)	0.093	**1.18 (1.04–1.35)**	**0.013**
AA	23/16	**2.13 (1.11–4.06)**	**0.022**	20/24	**1.96 (1.05–3.65)**	**0.034**	**2.04 (1.30–3.20)**	**0.002**
Dominant		**1.23 (1.03–1.47)**	**0.023**		**1.21 (1.01–1.45)**	**0.037**	**1.22 (1.08–1.39)**	**0.002**
Additive		**1.25 (1.06–1.47)**	**0.008**		**1.22 (1.04–1.44)**	**0.016**	**1.24 (1.10–1.39)**	**<0.001**

^a^Major allele (G) of rs1892901 was recognized as reference allele;

^b^Combined results were calculated based on meta-analysis based method(fixed effect model);

^c^Logistic regression with adjustment for age, sex, smoking and drinking status.
