# Supplementary material for: A functional SNP rs1892901 in FOSL1 is associated with gastric cancer in Chinese population
Source: Sci Rep. 2017 Feb 7;7:41737. doi: 10.1038/srep41737 (PMC5294397; doi:10.1038/srep41737)
Supplement: Supplementary Tables [file srep41737-s1.doc]

**SUPPLEMENTAL MATERIALS**

**A functional SNP rs1892901 in *FOSL1* is associated with gastric cancer in Chinese population**

Wenjie Liu, Tian Tian, Li Liu, Jiangbo Du, Yayun Gu, Na Qin, Caiwang Yan, Zhaoming Wang, Juncheng Dai, Zhining Fan

**Supplementary Table 1. Selected characteristics between gastric cancer cases and controls**

| Variables | Case (N = 1,140) | Control (N = 1,547) | *P* ^a^ |
| --- | --- | --- | --- |
|  | n (%) | n (%) |  |
| Age (yr) | 60.99 ± 10.54 | 60.68 ± 9.21 | 0.421 |
| Gender |  |  |  |
| Male | 862 (75.61) | 1153 (74.53) | 0.522 |
| Female | 278 (24.39) | 394 (25.47) |  |
| Smoking status |  |  |  |
| Never | 595 (52.19) | 812 (52.49) | 0.879 |
| Ever | 545 (47.81) | 735 (47.51) |  |
| Drinking status |  |  |  |
| Never | 679 (59.56) | 954 (61.67) | 0.269 |
| Ever | 461 (40.44) | 593 (38.33) |  |

^a^ T test was used for age and *χ^2^* test was used for other binary variables.

**Supplementary Table 2. Stratiﬁed analysis on the association of rs1892901 in *FOSL1* with gastric cancer risk based on the combination of the two stages**

| Characteristics | Case ^a^  (N = 2,146) | Control ^a^  (N = 3,820) | OR(95%CI) ^b^ | *P* ^b^ | *P_heterogeneity_* ^c^ |
| --- | --- | --- | --- | --- | --- |
| Age (yr) |  |  |  |  |  |
| <60 | 765/236/24 | 1388/368/20 | **1.22(1.04-1.44)** | **0.016** | 0.908 |
| ≥60 | 836/266/19 | 1597/425/20 | **1.21(1.03-1.42)** | **0.019** |
| Gender |  |  |  |  |  |
| Male | 1169/376/31 | 2217/603/29 | **1.23(1.07-1.40)** | **0.002** | 0.874 |
| Female | 432/126/12 | 768/190/11 | 1.20(0.94-1.52) | 0.135 |
| Smoking status |  |  |  |  |  |
| Never | 912/282/28 | 1510/398/20 | **1.27(1.09-1.49)** | **0.003** | 0.762 |
| Ever | 689/220/15 | 1475/395/20 | **1.23(1.03-1.45)** | **0.019** |
| Drinking status |  |  |  |  |  |
| Never | 1039/313/35 | 1701/469/20 | **1.22(1.06-1.41)** | **0.006** | 0.783 |
| Ever | 562/189/8 | 1284/324/20 | **1.26(1.05-1.52)** | **0.014** |

^a^ Major homozygote/heterozygote/Rare homozygote;

^b^ Derived from additive model using logistic regression analysis with an adjustment for age, sex, smoking and drinking status;

^c^ *P* for heterogeneity.

**Supplementary Table 3. Stratiﬁed analysis on the association of rs637571 in *FOSL1* with gastric cancer risk based on the discovery stage**

| Characteristics | Case ^a^  (N = 1,140) | Control ^a^  (N = 1,547) | OR(95%CI) ^b^ | *P_heterogeneity_* ^c^ | *P_interaction_* ^d^ |
| --- | --- | --- | --- | --- | --- |
| Age (yr) |  |  |  |  |  |
| <60 | 296/160/19 | 434/280/39 | **0.80(0.65-0.98)** | 0.277 | 0.427 |
| ≥60 | 416/218/28 | 484/267/37 | 0.93(0.77-1.11) |
| Gender |  |  |  |  |  |
| Male | 542/284/35 | 685/411/54 | 0.89(0.76-1.04) | 0.581 | 0.738 |
| Female | 170/94/12 | 233/136/22 | 0.81(0.60-1.08) |
| Smoking status |  |  |  |  |  |
| Never | 376/194/23 | 484/281/42 | 0.87(0.72-1.05) | 0.702 | 0.854 |
| Ever | 336/184/24 | 434/266/34 | 0.92(0.75-1.12) |
| Drinking status |  |  |  |  |  |
| Never | 422/229/25 | 565/340/45 | 0.88(0.74-1.05) | 0.953 | 0.951 |
| Ever | 290/149/22 | 353/207/31 | 0.89(0.72-1.10) |

^a^ Major homozygote/heterozygote/Rare homozygote;

^b^ Derived from additive model using logistic regression analysis with an adjustment for age, sex, smoking and drinking status;

^c^ *P* for heterogeneity；

^d^ *P* for interaction.
